# Porous Allograft Bone Scaffolds: Doping with Strontium

**DOI:** 10.1371/journal.pone.0069339

**Published:** 2013-07-26

**Authors:** Yantao Zhao, Dagang Guo, Shuxun Hou, Hongbin Zhong, Jun Yan, Chunli Zhang, Ying Zhou

**Affiliations:** 1 Department of Orthopedics, First Affiliated Hospital of the General Hospital of Chinese People’s Liberation Army, Beijing, China; 2 State Key Laboratory for Mechanical Behavior of Materials, Xi’an Jiaotong University, Xi’an, China; 3 Department of Prosthodontics, School of Stomatology, Fourth Military Medical University, Xi’an, China; University of Akron, United States of America

## Abstract

Strontium (Sr) can promote the process of bone formation. To improve bioactivity, porous allograft bone scaffolds (ABS) were doped with Sr and the mechanical strength and bioactivity of the scaffolds were evaluated. Sr-doped ABS were prepared using the ion exchange method. The density and distribution of Sr in bone scaffolds were investigated by inductively coupled plasma optical emission spectrometry (ICP-OES), X-ray photoelectron spectroscopy (XPS), and energy-dispersive X-ray spectroscopy (EDS). Controlled release of strontium ions was measured and mechanical strength was evaluated by a compressive strength test. The bioactivity of Sr-doped ABS was investigated by a simulated body fluid (SBF) assay, cytotoxicity testing, and an *in vivo* implantation experiment. The Sr molar concentration [Sr/(Sr+Ca)] in ABS surpassed 5% and Sr was distributed nearly evenly. XPS analyses suggest that Sr combined with oxygen and carbonate radicals. Released Sr ions were detected in the immersion solution at higher concentration than calcium ions until day 30. The compressive strength of the Sr-doped ABS did not change significantly. The bioactivity of Sr-doped material, as measured by the *in vitro* SBF immersion method, was superior to that of the Sr-free freeze-dried bone and the Sr-doped material did not show cytotoxicity compared with Sr-free culture medium. The rate of bone mineral deposition for Sr-doped ABS was faster than that of the control at 4 weeks (3.28±0.23 µm/day vs. 2.60±0.20 µm/day; *p*<0.05). Sr can be evenly doped into porous ABS at relevant concentrations to create highly active bone substitutes.

## Introduction

The long recovery time for bone defect healing highlights the need for a superior osteogenic scaffold [1,2]. Improving the osteogenic potential of implantable scaffolds will effectively shorten recovery time and provide a robust solution to critical sized bone defects, while reducing the occurrence of nonunion [3].

Elemental strontium (Sr) has recently gained attention for its ability to promote bone formation. Sr has been incorporated into hydroxyapatite (HA), tricalcium phosphate (TCP), and calcium phosphate cement (CPC) to improve their bioactivities and physicochemical properties [4-7]. Among various materials used as substitutes for autograft bone, freeze-dried allograft bone provides a natural scaffold for osteoconduction. Incorporation of Sr into allograft bone is an attractive method for developing a superior osteogenic scaffold. The application of Sr to allograft bone is comparatively safe and practical when compared with other cytokines [8]. However, the method for Sr incorporation is very specific for bone scaffold. For allograft bone, Sr cannot be mixed into the raw materials or added to the structure from the beginning. Furthermore, allograft bone cannot withstand any extreme conditions during preparation.

Sr can be detected in the mineral phase of natural bone, especially in regions of high metabolic turnover [9]. Biological apatites are known to have the capacity to exchange ions with the surrounding fluids [10], and bone tissue is a reservoir of mineral ions, capable of both binding and releasing them as needed for different biological functions. For example, calcium (Ca) ions in bone can be exchanged with other divalent cations in serum, such as Sr. When strontium ranelate (SrR) was administrated to treat osteoporosis, Sr deposition was targeted in bone tissue and promoted bone formation while inhibiting local bone resorption [11,12]. Sr can promote bone formation even at low concentrations [7]. *In vitro* studies have shown that Sr can enhance osteoblast replication and incite bone formation [13]. Furthermore, Sr can balance the bone formation and resorption processes *in vivo* [14,15], which decreases the incidence of bone nonunion. The replacement of some Ca^2+^ ions by Sr^2+^ ions improves the mechanical properties and the dissolution of HA, although the mechanism of action has not yet been clarified. The use of the trace element Sr is an interesting alternative to growth factors to promote bone formation. Moreover, making use of the ion exchange processes that occur in biological bone tissue is a promising method that can be applied *in vitro* to prepare Sr-incorporated scaffolds.

Freeze-dried allograft bone has a reasonable three-dimensional structure and is rich in bioactive ingredients. However, the manufacturing processes, such as washing, degreasing and irradiation, are not favorable for new bone growth and bone defect restoration [16,17]. In this study, Sr was incorporated into freeze-dried bone scaffolds using an *in vitro* ion exchange method to prepare a highly bioactive scaffold material. The effects of Sr-incorporation on scaffold physicochemical and mechanical properties, bioactivity, cytotoxicity, and bone formation rate were investigated.

## Materials and Methods

Allograft bone was provided by Xinkangchen Inc. (Beijing, China) and processed according to basic standard procedures, including hypothermal freezing, slicing into 0.2 g cubes with 5 mm sides, degreasing, freeze-drying, and sterilization.

Strontium chloride reagent SrCl_2_·4H_2_O (Shanghai, China) was dissolved in deionized water at 5, 10, 20, 30, and 40 mM and sterilized by autoclave at 128 °C for 30 min. Cubes of bone were immersed in the SrCl_2_ solutions at a mass ratio of 1:200 using aseptic technique. A vibration machine (THZ-82, Guohua, China) was used to promote the reaction at 25 °C. Vibration time lasted 14 days to achieve incorporation of Sr. Bone cubes were next ultrasonically washed with deionized water 4 times to remove residual SrCl_2_ solution. Cubes were then freeze-dried and sterilized for preservation.

### Inductively coupled plasma optical emission spectrometry

Freeze-dried bone cubes processed as described above were dissolved in 68% nitric acid. After 12 h, bone particles were completely dissolved and had a clean outer appearance. The Ca and Sr contents of bone samples were determined from these solutions by inductively coupled plasma optical emission spectrometry (ICP-OES) (IRIS Intrepid 2 XSP, USA). The Sr incorporation ratio, expressed as a molar ratio [Sr/(Sr+Ca)], was calculated to show the relative density of Sr in bone mineral.

### Energy-dispersive X-ray spectroscopy

Bone cubes were sliced into halves and the inner surface was coated with gold, and then examined by energy-dispersive X-ray spectroscopy system (JSM6460, JEOL, Japan). The route across the trabecular wall was selected for line scanning to analyze the distribution of elements of interest, including Sr and Ca.

### X-ray photoelectron spectroscopy

Portions of the samples were ground into powders and analyzed by X-ray photoelectron spectroscopy (XPS) (Thermo, Fisher, USA). Major parameters for analysis were: aluminum target, 100 W power, 10^-9^ Pa vacuum, 0.5 eV minimum energy resolution, and 30 µm minimum step size.

### Mechanical testing

Compressive testing on the bone cubes was performed by a multi-functional testing machine (AGS-500, Shimadzu Corp, Japan). Tests were carried out at a very low load speed of 0.5 mm/min to avoid viscoelastic effects of the bone scaffold. The compliance of the system was accounted for to give a true measurement of the bone displacement. To improve the reproducibility of the compressive test, a 10 cycle precycling load (up to 5 N) was first applied to the hydrolyzed specimens [18]. The strength-strain curves were recorded and the maximum strength and Young’s modulus of each sample were computed. Six samples for each group were tested and t-tests were used to assess the significance of differences between Sr-doped ABS and controls.

### Release curve

*In vitro* Sr^2+^ ion release was measured by placing one bone cube (0.125 cm^3^) into 20 mL of demineralized water. The immersion liquid was changed daily [19]. Released Sr^2+^ ions in the immersion liquid were separately measured by ICP-OES (IRIS Intrepid 2 XSP, USA) on days 1, 3, 5, 7, 14, 21 and 30.

### Bioactivity in simulated body fluid

Individual bone cubes were immersed in 120 mL of acellular simulated body fluid (SBF) with pH 7.40 and ionic concentrations: Na^+^ 142.0, K^+^ 5.0, Mg^2+^ 1.5, Ca^2+^ 2.5, Cl^-^ 147.8, HCO^3-^ 4.2, HPO_4_^2-^ 1.0, SO_4_^2-^ 0.5 mM, nearly equal to those in human blood plasma at 36.5 °C [20]. The SBF was prepared by dissolving reagent-grade chemicals of NaCl, NaHCO_3_, KCl, K_2_HPO_4_·3H_2_O, MgCl_2_·6H_2_O, CaCl_2_ and Na _2_SO_4_ (Nacalai Tesque Inc., Kyoto, Japan) into distilled water and buffering at pH 7.40 with tris (hydroxymethyl) aminomethane ((CH_2_OH)_3_CNH_3_) and 1.0 M hydrochloric acid (Nacalai Tesque Inc., Kyoto, Japan) at 36.5 °C. After being immersed in SBF for one week, samples were analyzed by JSM6460 scanning electronic microscopy (JEOL, Japan) to observe surface structure and morphology.

### Cytotoxicity analysis

Cytotoxicity assays were carried out following standard laboratory procedures according to the international standard ISO 10993-12 [14]. One gram of material, sterilized by gamma radiation, was placed into glass flasks, which were then loaded with 10 mL DMEM culture medium containing 10% fetal bovine serum (FBS) at pH 7.3. The flask was incubated in a humidified atmosphere of 5% CO_2_ at 37 °C for 48 h. The supernatant was then filtered through a membrane and dilutions were made at 100, 50, 25, and 10% pure extracts. A suspension of L929 cell was prepared at 2×10^4^ cells/mL and 100 µL of the suspension was added to each well of a 96-well tissue culture plate for cell adhesion. After the adhesion, the culture medium was removed and each dilution and the control culture medium (DMEM + 10% FBS) were added to the plate with 6 replicate wells per condition. The plate was incubated in a humidified incubator with 5% CO_2_ at 37 °C for 1, 3, and 5 days, after which 20 µL methyl thiazolytetrazolium (MTT) solution at 5 g/L was added to each well and incubation continued for 4 h. After this period, the supernatant of each well was carefully aspirated and 150 µL of dimethyl sulfoxide (DMSO) was added, followed by 5 min shaking. The optical density (OD) at 490 nm was read on a spectrophotometer (MR600, Dynatech), which was used to calculate the relative growth rate (RGR) of L929 cells according to the following equation: RGR (%) = OD _tested sample_ / OD_control group_.

### Bone formation rate

The *in vivo* bone formation rate for Sr-doped ABS was assessed by implantation in a rabbit tibial defect model. All animal procedures were approved and overseen by the Institutional Animal Care and Use Committee of First Affiliated Hospital of PLA General Hospital. Twelve healthy, skeletally mature New Zealand white rabbits (20 weeks old, 2.5 ± 0.3 kg) were included. Rabbits were anesthetized with intravenous sodium pentobarbital (50 mg/kg). The medial portion of the radius was exposed through a skin incision of approximately 3 cm in length and a bone defects was made by removing a 1.5 cm diameter bone section from the middle of the radius. Next, freeze-dried bone and Sr-doped bone powders were separately implanted to repair the defect. The surrounding tissues were sutured to keep the implants in position and the wound was closed according to standard operation procedures. Analgesia was provided by the administration of meloxicam at 0.2 mg/kg by intramuscular injection prior to surgery, followed by additional doses every 24 hours for 3 days. Food intake and body weight were recorded daily. Calcein was injected intramuscularly at a dose of 10 mg/kg on days 25 and 28. Three days after fluorescent labeling, animals were sacrificed by an intravenous overdose injection of pentobarbital. The middle section of the radius was taken out and prepared according to solid tissue slicing procedures. The slices obtained were observed with fluorescent microscopy and measured with Osteomeasure software (Military medical science research institute, China). T-tests were used to compare differences between groups.

## Results

### ICP-OES

**Figure 1:**
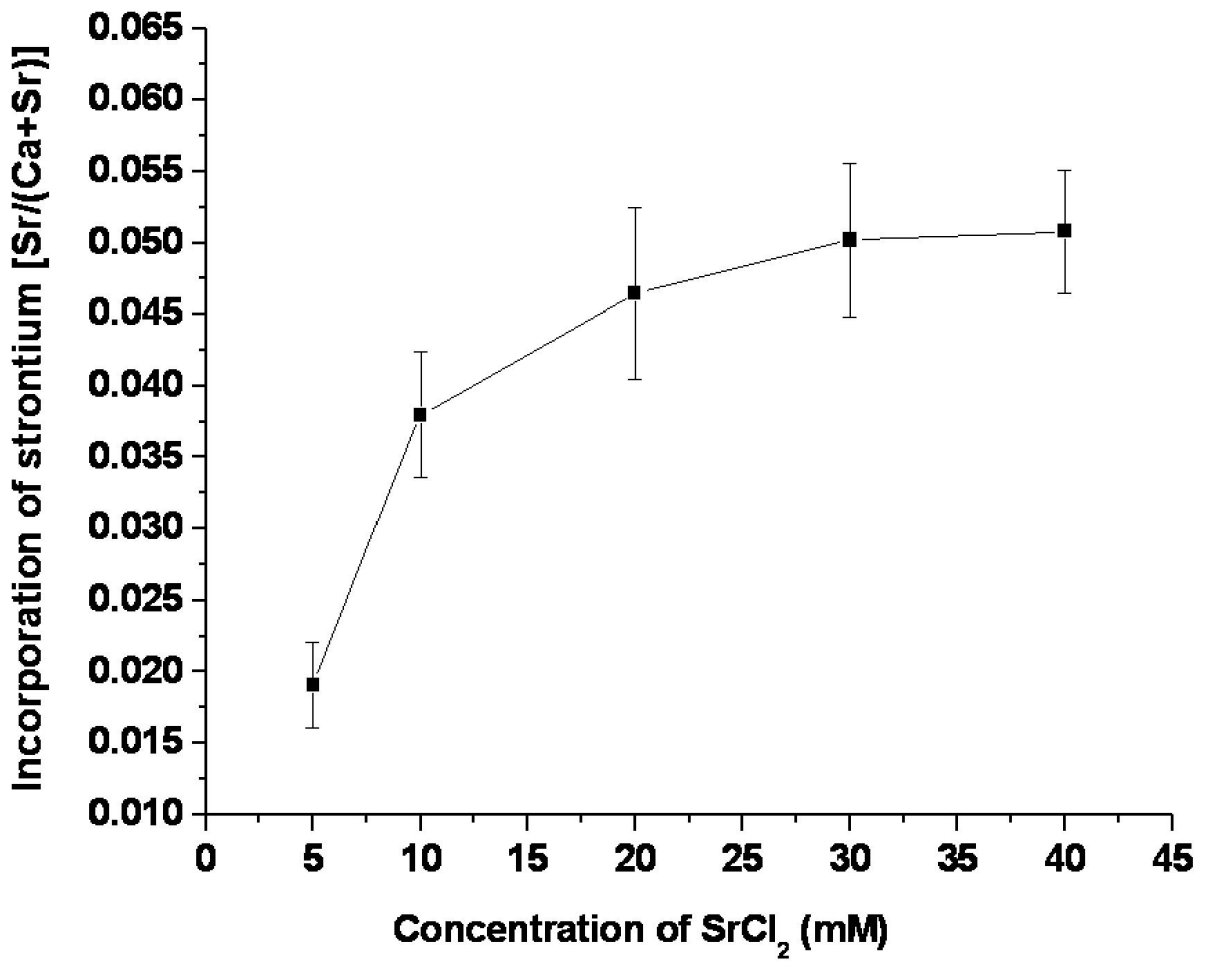
Incorporation of strontium in bone as a function of SrCl_2_ concentration in the immersion solution.

The allograft bone without treatment showed a Sr concentration approaching 0.01%. Sr concentration in Sr-doped ABS showed an increasing trend with the increase in SrCl_2_ concentration from 5 mM to 40 mM (Figure 1). When the concentration of the SrCl_2_ solution was above 30 mM, incorporated Sr density surpassed 5%.

### Line scanning

**Figure 2:**
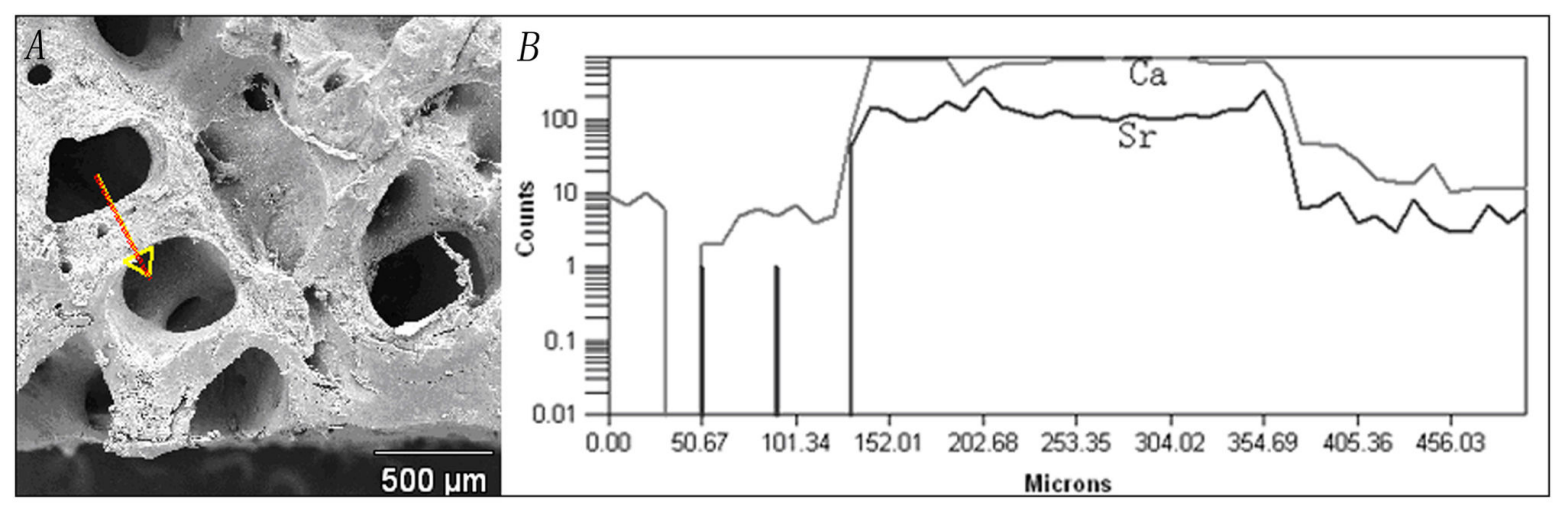
Line scanning route and distribution of Ca and Sr elements in ABS. A) The arrow shows the scanning route. B) The relative quantity of Ca and Sr elements across the scanning route. X axis represents the distance from the starting point and the Y axis represents the relative quantity of each element recorded during the examination.

Line scanning of the samples indicated that elemental Sr distributed comparatively evenly across the inner matrix of the trabecular wall (Figure 2). Within the bone mass, the density of Sr was nearly constant, whereas near the trabecular wall surface, the concentration of Sr was slightly higher.

### XPS

**Figure 3:**
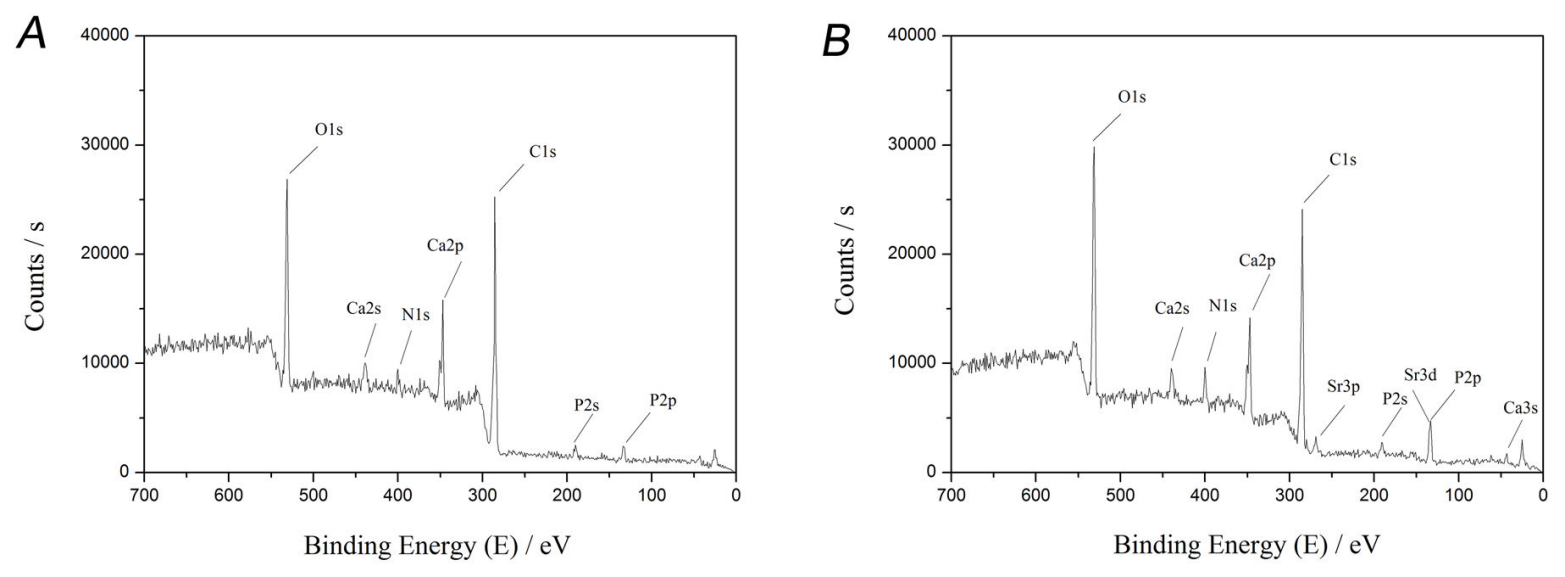
XPS analysis of bone powder: A) XPS result before treatment. B) XPS result after treatment. The peak binding energy at 269.13 eV is from Sr3p3/2, at 133.52 eV is from Sr3d5/2, and at 347.08 eV is from Ca2p3/2.

XPS results confirmed the incorporation of Sr into bone after SrCl_2_ solution treatment (Figure 3). Two major peaks for Sr were observed with binding energies of 269.13 eV (Sr3p3/2) and 133.52 eV (Sr3d5/2). As expected, the incorporation of Sr increased the Ca element binding energy, with the Ca2p3/2 peak increasing from 347.08 eV to 348.24 eV. In addition, single element analysis suggested that Sr mainly combined with oxygen atoms or carbonate groups, which is the same formula as how Ca exists in bone.

### Mechanical testing

The average compressive strength and Young’s modulus of the two groups are reported in Table 1. There were no significant differences for compressive strength and Young’s modulus between the two groups (*p* > 0.05).

**Table 1:** Compressive strength and Young’s modulus of the samples.

Samples	Maximum strength MPa (Mean ± SD)	Young’s modulus MPa (Mean ± SD)
ABS	4.2 ± 1.8	270 ± 66
Sr-ABS	4.5 ± 1.6	287 ± 73

### Ion release curve

**Figure 4:**
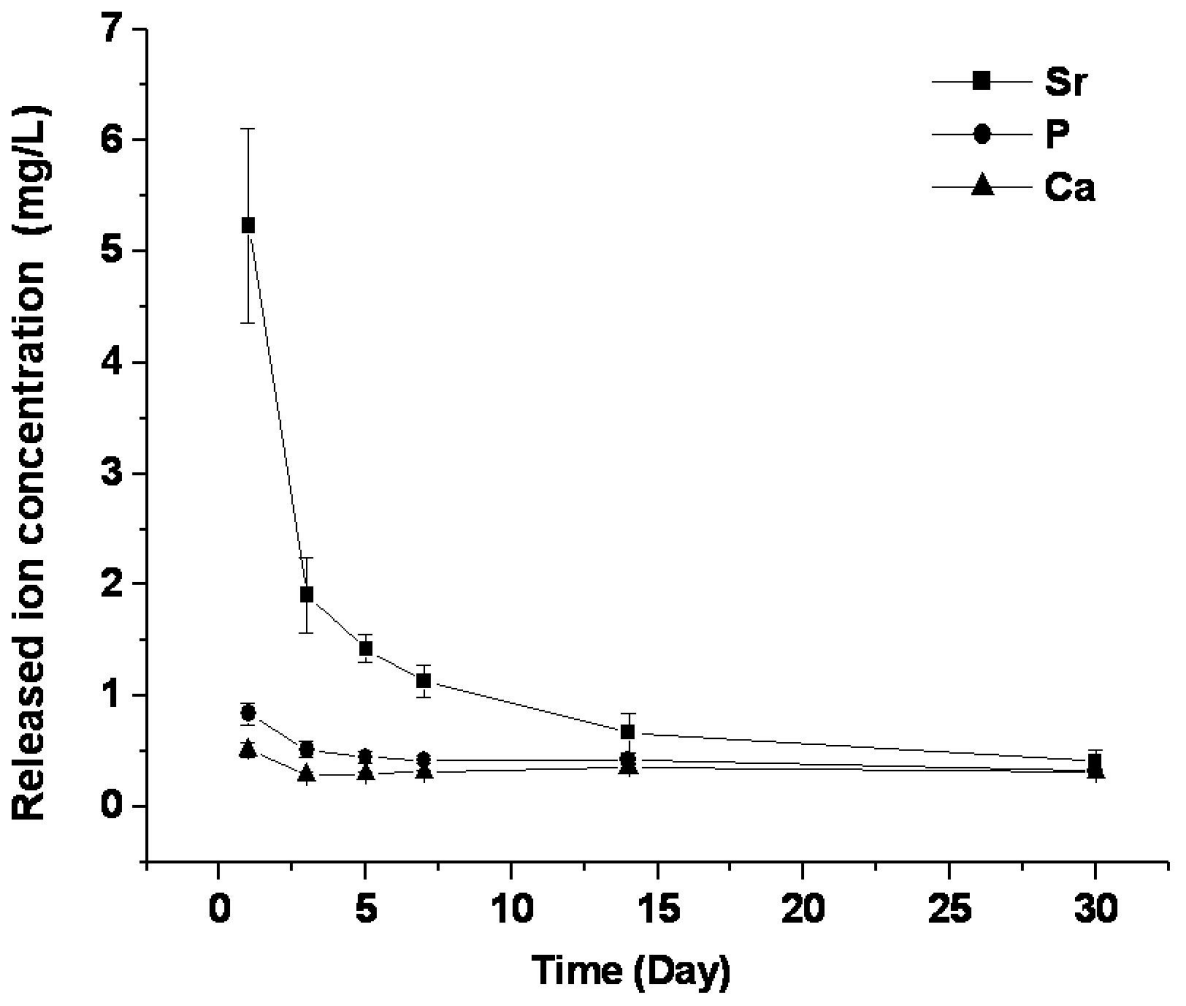
Release curve of strontium ions from Sr-doped ABS into deionized water.

The time-release profile of Sr is shown in Figure 4. Sr ions were detected in the immersion fluid from the beginning. The largest release of Sr occurred early, especially on the first day. As time progressed, the release of Sr ions decreased sharply, plateauing after 2 weeks, and approaching, though slightly higher than, the concentration of Ca ions released from bone on day 30 (*p* < 0.05).

### Biocompatibility via SBF test

**Figure 5:**
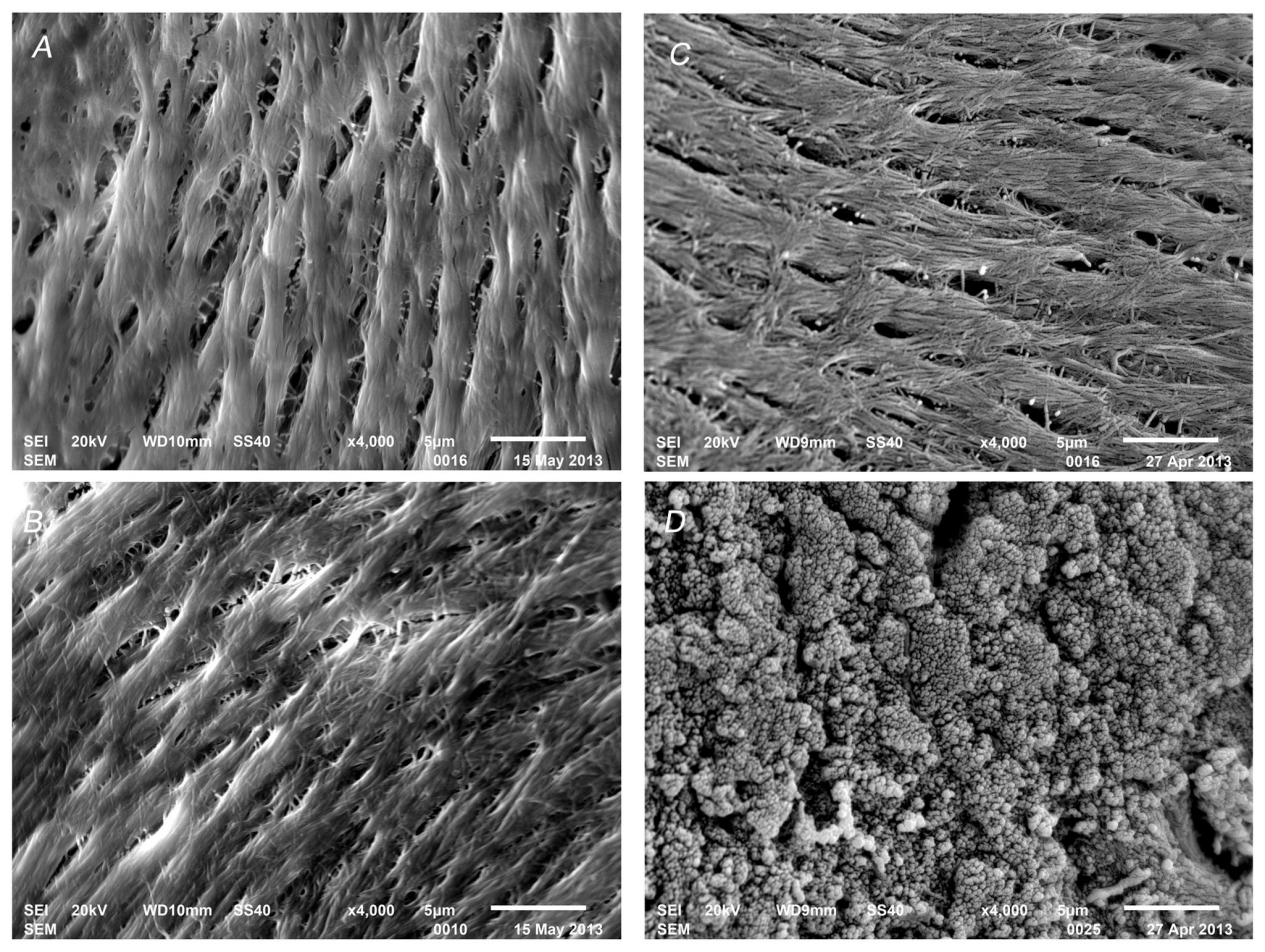
Surface analysis of samples by SEM before and after soaking in SBF. A) freeze-dried ABS before soaking. B) Sr-doped ABS before soaking. C) freeze-dried ABS after soaking. D) Sr-doped ABS after soaking.

Before soaking in SBF, both groups of bone scaffold exhibited a clean surface with a few scattered mini-pores. After soaking, few small bone-like apatite particles were found on the surface of the bone scaffolds, while larger quantities of bone-like apatite were found on the surface of Sr-incorporated samples (Figure 5).

### Cytotoxicity

**Figure 6:**
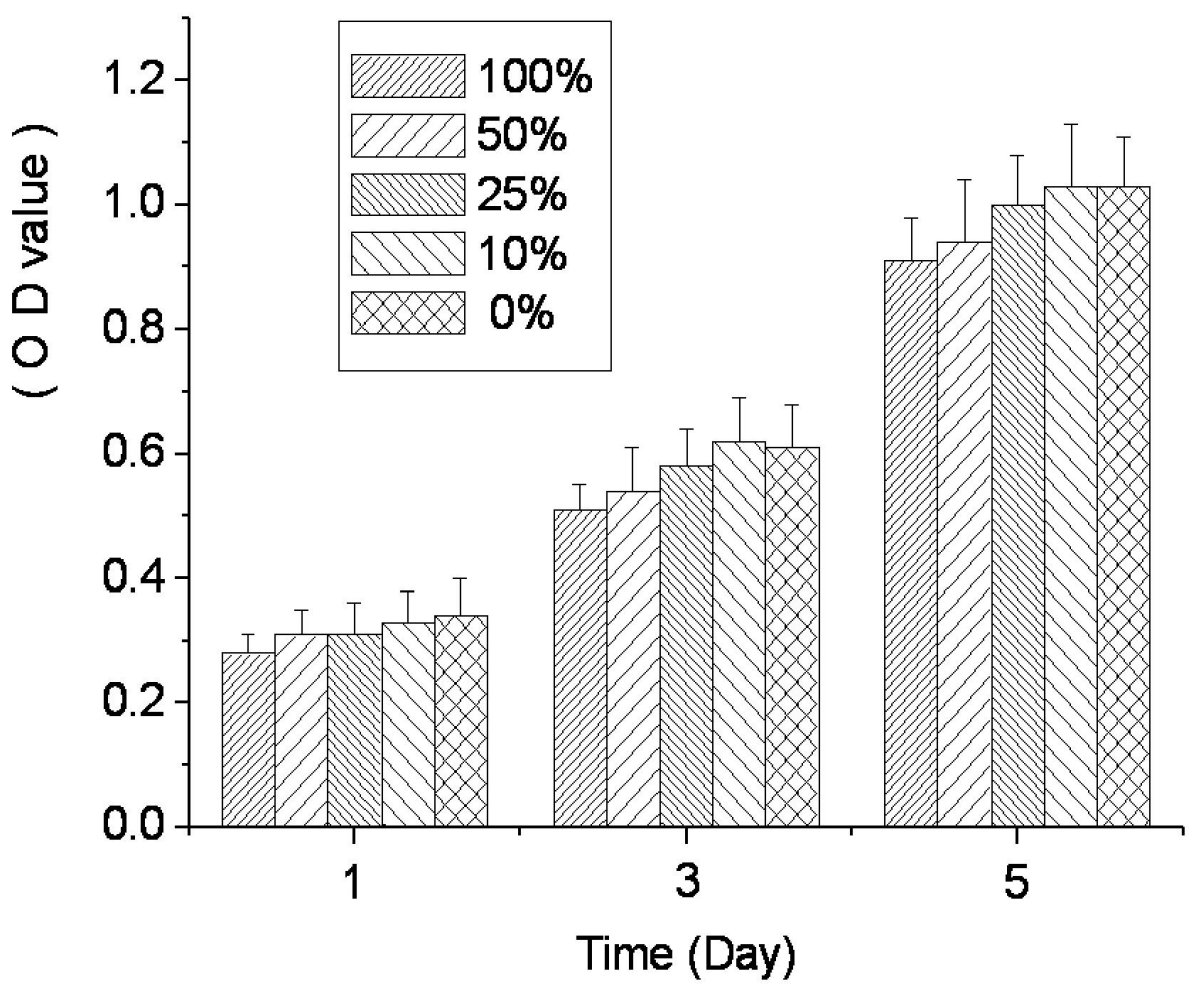
RGR of L929 cell showed as OD values in the cytotoxicity test of Sr-doped ABS.

Cytotoxicity, as measured by cell growth shown via MTT OD, is shown in Figure 6. On the first day, OD values for all groups were comparatively low, but increased over time. On day 3, the OD values for each group had doubled compared with day 1. On day 5, the OD values were above 1.0 for the 10% extract and DMEM groups. The RGR of each concentration group calculated by MTT OD were all above 75%.

### Bone formation rate

**Figure 7:**
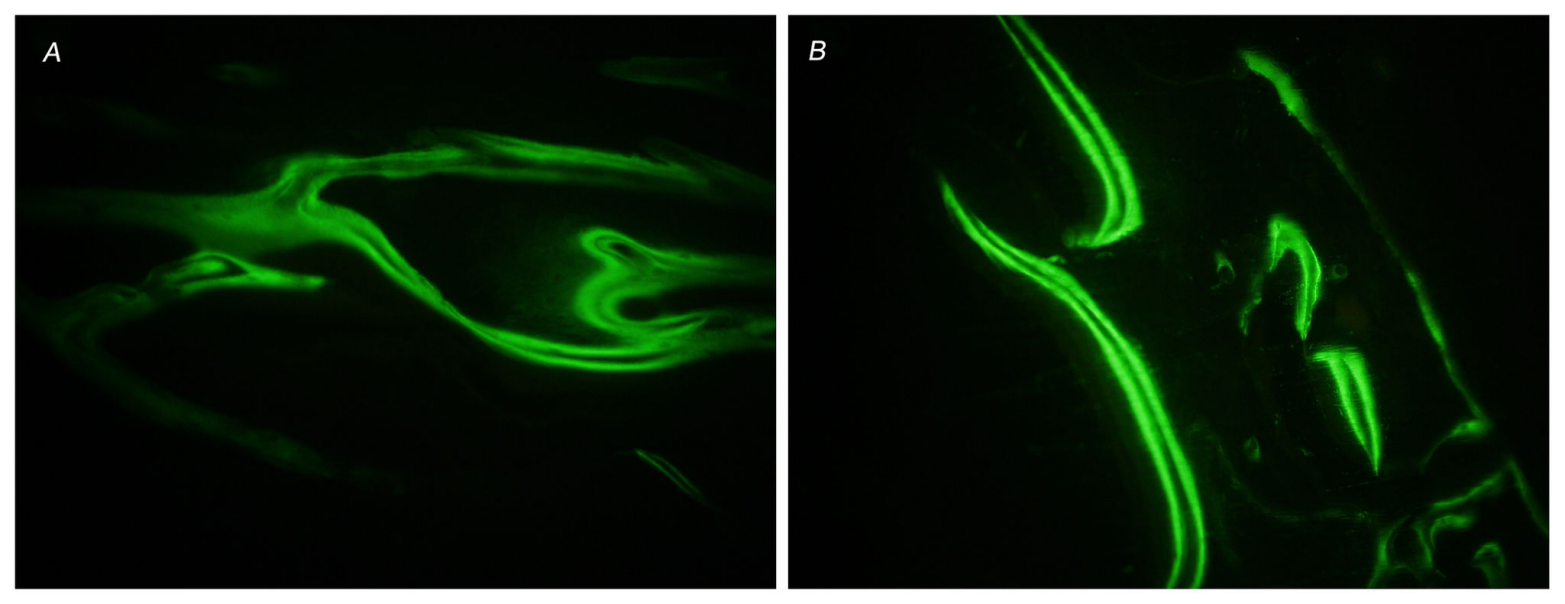
Double lines of fluorescent labels showing bone mineral deposition interval in *in vivo* test. A) Control group with a bone mineral deposition rate of 3.26±0.50 µm/day. (×200). B) Sr-doped ABS group with a bone mineral deposition rate of 4.02±0.45 µm/day. (×200).

Robust Ca deposition was observed for both groups as shown in Figure 7. Adjacent double lines in the specimen indicated the mineral deposition interval between the two fluorescent label time points. The rate of new bone formation of Sr-doped ABS was significantly higher than that of control group at 4 weeks (3.28±0.23 µm/day vs. 2.60±0.20 µm/day, *p*<0.05).

## Discussion

Sr is a natural bone-seeking trace element, which resembles Ca chemically and physically, that preferably accumulates in new trabecular bone depending on the site within the skeleton. In particular, the Sr content in a new compact bone is 3 to 4 fold higher than that in existing compact bone, and approximately 2.5 fold higher in new than in existing cancellous bone [12,21]. The Sr concentration in native trabecular bone in the current study was found to be less than 0.01%, similar to previous reports [22], which is negligible compared with the incorporation levels attained here.

Sr is mainly incorporated into the mineral crystal *in vitro* by ionic substitution of Ca. XPS indicated two major peaks for Sr with binding energies of 269.13 eV (Sr3p) and 133.52 eV (Sr3d). Incorporation of Sr induced a slight alteration of Ca element binding energy. In addition, further analysis suggested that Sr mainly combined with oxygen atoms and carbonate groups, which is the same formula as how Ca exists in bone. These results suggest that Sr entered the bone tissue via the ion exchange mechanism. Low-temperature exchange reactions have been described elsewhere [23], and they generally occur in aqueous media and in most instances involve a dissolution–reprecipitation mechanism. With regard to the dissolution–reprecipitation mechanism primarily occurring on the scaffold surface, it is quite reasonable that the Sr concentration of the outer surface is higher than the inner surface. The effective ion exchange deep inside the bone mass was possibly due to the numerous biological pores, as shown in SEM photographs, and the existence of hydrophilic collagen structures in bone tissue.

At high doses of Sr by oral administration [21], molar ratios [Sr/(Sr+Ca)] in the range of 0.5–3% were found in cynomolgus monkey bone after 13 weeks of treatment [12]. The molar ratio of Sr in the current study by far surpassed the data achieved within 13 weeks under the normal orally administered dose. A different investigation reported on bone material quality in transiliac bone biopsies from postmenopausal osteoporotic women treated longer than 3 years with Ca and vitamin D plus either 2g SrR per day or placebo [24]. The authors observed incorporation of Sr in bone tissue up to 6% [Sr/(Sr+Ca)], similar to the level achieved in the current study. *In vivo*, doped Sr levels correlated with the SrR serum levels of the individuals. *In vitro*, doped Sr levels were also mostly directly related to the concentration of the SrCl_2_ treating solution, while above 20mM the curve increased gently.

Ion exchange has been previously reported to be undetectable in porous samples of stoichiometric HA prepared by the impregnation of cellulosic sponges with a suspension of stoichiometric HA powder, successive sintering at 1250 °C, and immersion in solutions of 0.5 and 1 M Sr(NO_3_)_2_ solution for up to 5 days [23]. In this case, few Sr ions were found to have substituted for Ca in the porous apatite samples. This lack of ion exchange could be attributed to the low reactivity of the sintered HA samples. In fact, the specific surface areas, the material surfaces available for ion exchange, are very different between the collagen interconnected natural bone and the so-called porous sintering HA scaffolds.

Nanocrystalline apatites offer faster reaction times and improved capabilities for ion exchange compared with well-crystallized apatites [23]. The nano-effect of particles is mainly due to dimensional factors and boundary effects that create unusually high surface energy and reactivity. Bone apatite is known to consist of hydroxyapatite (HA) with nano-crystals [25]. The nanostructure of the bone scaffolds likely contributed to the high ion exchange rate of Sr for Ca in the current study. Biological apatite, consisting of nano-crystals, is very active and contains more exchangeable ions than synthesized HA [26].

Furthermore, natural bone apatite contains other components, such as carbonate groups [27]. In the solution environment, divalent metal ions can combine with carbonate groups to form insoluble products. The solubility product equilibrium constants (Ksp) for the reaction follow the order: MgCO_3_ > CaCO_3_ > SrCO_3_ [28]. This order suggests that carbonated components are apt to form SrCO_3_ in the presence of Sr ions. The carbonate quantity of the biological apatite is above 2 wt%, which has a large potential to influence incorporation of Sr into bone scaffolds [26,29].

In one study of the trace metals present in human bone, Sr was the only one that was correlated with bone compression strength [30]. Sr has also been found to show anti-fracture efficacy in the treatment of postmenopausal osteoporosis [31,32]. The dissolution-reprecipitation mechanism that occurs is apt to repair defects on the bone scaffold surface, which is helpful for compressive resistance. In the current study, the Sr-doped ABS did not show a significant improvement in the compression strength over controls. However, it did not reduce the strength of ABS either.

Incorporation of Sr can make ABS more advantageous, such as by contributing to the bone forming process through local release of Sr. Therefore, the release profile of Sr from the Sr-doped ABS was determined. Landi et al. [26] synthesized a Sr-substituted HA with 8.7 wt% Sr content and measured the *in vitro* Sr^2+^ ion release by immersing 3 g of HA granules in 50 ml of Hanks’ balanced salt solution with 6.060 ppm Sr^2+^ (6.060 mg/L). After 24 h, the Sr concentration decreased to only 0.602 ppm on day 7 with daily changes of the immersion liquid. In the current study, a mass to liquid ratio of 1:200 was used, which achieved much higher Sr^2+^ concentrations in the immersion liquid. Even low doses of Sr^2+^ released from bone cement can influence cellular response, as was shown for an α-TCP-based cement/gelatin composite with 5 mol% Sr element [33]. Release of Sr from the local defect site can promote cell function directly and avoid the potential deleterious effects of oral administration resulting from the reduction in the intestinal absorption of Ca [7].

A burst release of Sr from Sr-doped ABS was observed. In addition, Ca and phosphate elements both have similarly high initial burst release rates, indicating that the freeze-drying procedure might partially solubilize biological apatite. Unstably incorporated Sr in the apatite crystal is apt to leak out during early time points, when the environmental Sr concentration dropped sharply. During the initial stage, the quantity of phosphate in the immersion fluid was twenty-times less than the total amount of Sr and Ca. Over time, the (Ca+Sr)/P ratio decreased, approaching 1.67. Unstably incorporated Sr may explain this release profile, since some fraction of the Ca or Sr positions in HA crystal are more apt to be ionized and exchanged, as mentioned previously. The Ca at these positions had mostly been substituted by Sr, and thus the Ca element released at the initial time was even lower than phosphate release. The existence of SrCO_3_ might contribute to the high Sr release rate at the initial time point. The solubility of SrCO_3_ is 0.011 g/100 mL at 18 °C, which is much higher than the solubility of HA (0.4 ppm). The high initial release rate of Sr could be helpful to quickly increase the concentration of Sr ions at a wound site. The bioactivity of a bone substitute material is often evaluated by examining the capability of bone-like apatite formation on its surface in SBF with ion concentrations nearly equal to those of human blood plasma. Although the efficacy of this method for evaluating bone-bonding ability is still debated, the formation of bone-like apatite on the surface of biomaterials can be regarded as a preliminary indication of their biological activity [34]. The results here suggest a beneficial interaction between the Sr-doped ABS and body fluid.

The cytotoxicity analysis demonstrated that L929 cells grew robustly, even in the presence of 100% extract from Sr-doped ABS. The RGRs of all concentration groups were above 75%, and the Sr-doped ABS was ranked ‘1st class’, meaning the material is non-toxic. OD values in the 10% extract group were slightly higher than in the 100% extract group, possibly owing to the higher ion concentrations and osmotic pressure in the 100% extracts. Alternatively, the slight growth inhibition with high extract concentration might be due to the chelation of potentially harmful elements by fetal bovine serum proteins. However, according to the cytotoxicity standard 75% RGR, all groups are safe and non-toxic. The results suggest that any potentially harmful elements of the Sr-doped ABS were within safe concentration ranges and the scaffold is biocompatible with cultured cells. Furthermore, the in vivo results further support the biocompatibility and safety of the Sr-doped ABS with direct evidence: robust bone formation. If deleterious effects of Sr were observed after further study, the doping ratio can easily be lowered from 5% by changing the solution concentrations (Figure 1).

The *in vivo* results indicated a higher bone mineral deposition rate and more active positions on the bone formation interface in the Sr-doped ABS. Other studies have investigated the effects of Sr-substituted hydroxyapatite coatings on osseointegration and implant mechanical fixation [35,36,37,38], demonstrating that SrHA coatings enhance implant osseointegration. In the current study, the Sr incorporation was effective in enhancing bone formation rate and the incorporation ratio approached the Sr-doped level in bone after long-term oral administration of SrR [24]. The biocompatibility of the Sr-doped ABS material is satisfactory. Therefore, the Sr-doped ABS might be a safe and more effective material for the treatment of bone defects.

## Conclusion

Sr can be incorporated into ABS at a molar density greater than 5% [Sr/(Sr+Ca)] by immersion in SrCl_2_ solution. Sr distributes comparatively evenly in bone scaffolds and released Sr ions can be detected for 4 weeks. Sr-doped ABS displays satisfactory safety and improved bioactivity in both *in vitro* and *in vivo* experiments.
